# A Practical Treatise on the Diseases of Children

**Published:** 1844-08

**Authors:** 


### REVIEWS.

---

Art. V.—*A Practical Treatise on the Diseases of Children.* By D. Francis Condie, M. D., Fellow of the College of Physicians; Member of the American Philosophical Society; Honorary Member of the Philadelphia Medical Society, &c. Philadelphia: Lea & Blanchard. 1844. 1vol. 8vo. pp. 651.

Books on the diseases of children are multiplying almost as rapidly as the children themselves—at least

Every day and year brings forth a new one; and if the rate of increase continues, nothing short of Malthus' proposition will put a stop to it. What with out-and-out new ones, and old ones renewed, '*rifaciamento'd*,' revamped—a modicum of new wine put into old bottles—we bid fair to have about as many of the books as of the children. The press is 'prolific,' truly, *quoad hoc*, not to mention the foreign ones Americanized or adopted: for, as in the glorious days of 'rare Ben Jonson,' it was the custom for extraordinary men to have adopted sons, spirits congenial to their own; so in these latter times, our extraordinary medical authors adopt and christen with their name the mental offspring of others. Whether with the books, as with the 'human blossom,' every change shows some new beauty, we are not prepared to say.

Brodie somewhere observes, in relation to a *plaster*, got up by an old fellow named Kirkland, whose name it bore, that how it should have ever entered into any man's head to make such a composition, he does not know; but the composition being invented, he is bound to say it is a very useful one. Something similar to this may be said of Dr. Condie's book. How he should have conceived it *necessary* to make the book, we do not know; but being made, we must say, we believe it to be a very good one.

We do not intend, nor is it indeed necessary, to give a lengthy analysis of this book—its nature forbids the attempt. We plunge therefore at once into the middle of the volume, selecting such matters as seem to us novel, or as will serve to give our readers a touch of the author's quality.

Our first extract will relate to what we apprehend is very rare—*polypus of the rectum.*

"Polypus in this situation, has been very generally overlooked, it being, probably, mistaken for prolapsus ani, to which it bears a very close resemblance. It is, nevertheless, somewhat remarkable, that it should have escaped the notice of almost every modern writer on the diseases of children;—for, although of not very frequent occurrence, it is, nevertheless, much more so than physicians would appear to be aware of. Several instances have fallen under our notice; and although they were generally presented to us as cases of prolapsus ani, yet, upon a careful and minute examination,—a neglect of which, in such cases, would be unpardonable—we have never found the least difficulty in detecting the true character of the complaint.

"When a polypous tumor forms in the rectum, during childhood, the little patient is troubled, at intervals, with a repeated, often ineffectual desire to evacuate the bowels, generally attended with considerable straining, during which, sooner or later, and finally, every time the straining recurs, a tumor is protruded from the anus—varying in size in different cases, from that of a cherry, to that of a large hickory-nut. The tumor is, usually, of a bright, or dark red color, but often white, or of a dirty yellow; it is, in most cases, thickly covered with a tenacious, bloody mucus. When of a dark red, or purple hue, it is apt to bleed freely, especially when handled or irritated. When the tumor is protruded, it is situated in the centre of the anus, and entirely without the sphincter, and appears as if it were attached, all around, to the edge of the anus. On passing the finger into the rectum, a slender pedicle is found to proceed from the base of the tumor, for a short distance within the gut, to the inner surface of which it is attached. When the polypus is of considerable size, the straining efforts to evacuate the bowels are often very violent, and attended, sometimes, with considerable pain; and when it is finally protruded, it is found to be surrounded by a slight eversion of the lower portion of the rectum. In one of the cases that came under our notice, the tumor, which was of large size, separated, and came away spontaneously; the hæmorrhage which followed was very slight, and soon ceased, without the necessity of our resorting to even a compress. In the other cases, the tumors were readily removed by ligatures—in the application of which there is not the least difficulty—without the occurrence of any severe or untoward symptom."

In looking over the chapter on the diseases of the respiratory organs, the term *atelectasis pulmonum* arrested our attention. Dr. Condie gives the following account of it from Jörg:

"The disease consists in an agglutination or obliteration of the pulmonary cells, to a greater or less extent, in one or both lungs. The portion of the lung in which this obliteration of the cells exists, is depressed below the level of the surrounding tissue, and is of a dark red or violet hue. It presents, when incised, a smooth, red surface, from which a bloody serum may be squeezed, but in which no air bubbles can be detected. This condensation of the tissue of the lung, from obliteration of the air cells, is not produced by either inflammation or effusion, but is the result of imperfect respiration, which prevents the air from penetrating and distending all the cells of the lungs; the parts affected, consequently, retaining the color and density of the fœtal lung, and sink when placed in water.

"The causes assigned for this affection, are, a very rapid and easy delivery, or a too strong compression of the head of the child during parturition; both of which circumstances are common causes of asphyxia in new-born infants; or when respiration does take place, prevent it from being sufficiently full to dilate the whole of the texture of the lungs, and, hence, give rise to the disease under consideration.

"The affection is especially characterized by an imperfect, short, anxious, and sometimes scarcely perceptible or intermittent respiration; a feeble, plaintive cry, difficulty of sucking, an imperfect elevation of the ribs and sternum, livid or blue color with coldness of the skin, a weak languid pulse, and symptoms of general prostration. In consequence of the imperfect respiration, and impeded circulation, nutrition is always impaired, and hence, if the child survive, it becomes emaciated, and cannot bear the slightest motion or exercise; in some cases congestion of the brain is produced, and convulsions; or, from the violence of the respiratory efforts, inflammation of the bronchii and lungs.

"The efforts of the obstetrician should be, as early after delivery as possible, to induce in the infant a deep, full, vigorous inspiration; for, if the respiration be allowed to continue weak, and the lungs to be but imperfectly expanded, the infant seldom survives for any length of time. The mouth of the infant should, therefore, be cleared of any mucus that may be present in it; and by smart slaps upon the glutei muscles, or upon the palms of the hands and feet, respiration will often be fully established, or we may proceed as directed in the section on asphyxia. Rubbing the chest and back with sulpuric ether, and introducing it into the nostrils and mouth, immersion in an aromatic warm bath, and repeated clysters and emetics are recommended by Jörg, all of which are of doubtful propriety. Emetics, and all means which tend to increase the engorgement of the vessels of the brain, are, indeed, strongly contra-indicated when the brain has been injured during labor; in these cases a few grains of calomel, and stimulants to the lower extremities, cool lotions to the head, and even leeches to the latter, will be found of advantage."

Under the head of *laryngismus stridulus—spasmodic croup*, our author treats of the affection that has recently attracted attention under the name of thymic asthma. Kopp, of Germany, was the first who accurately described this disease, attributing it to hypertrophy of the thymus gland, an opinion which has given rise to considerable discussion, and is upheld by many German and British practitioners. The prevailing sentiment, which is also that of our author, is that the enlargement of the gland is merely accidental, and has nothing at all to do with the essential symptoms of the disease, being rather a consequence than a cause.

In the section on diseases of the nervous system, Dr. C. expresses the belief that *cerebral apoplexy* and *paralysis* are more frequent in children than is generally supposed. He states in a note that, in Philadelphia, during the 35 years preceding 1842, there were 67 deaths from apoplexy; of which 27 were under 1 year, and 16 between 1 and 2 years. He thinks that a large proportion of those reported as dying of convulsions, disease of the brain, and acute hydrocephalus, really fall victims to apoplexy. Of the symptoms he says:

"The attack is generally sudden; but, in many cases, it may be preceded for some days by a deranged condition of the bowels; or it may occur after an attack of convulsions, or in the course of some other disease. The symptoms are, invariably, more or less complete stupor, with a tumid and livid appearance of the face, contraction and insensibility of the pupils, laborious or stertorous respiration, and, occasionally, convulsions, or a spastic rigidity of the neck and lower extremities. On recovering from the state of stupor, the child may exhibit no lesion of motion or sensation on either side of the body, or one side, or the upper or lower extremity of one or the other side, may be in a state of complete or partial paralysis."

The prognosis of this state is not so unfavorable, if proper measures are promptly resorted to. If, however, it is allowed to run on, or if the attacks are repeated at short intervals, effusion on the surface of the brain, or some structural lesion is likely to occur, ending in death, or in greater or less impairment of intellect. It is most manageable in very young children, least so in the older.

The morbid appearances usually found, are engorgement of the cerebral vessels and sinuses, and sanguinous oozing from small bloody points upon the medullary surface when cut, occasionally serous effusion under the arachnoid, at the base of the brain, in the ventricles or theca of the spinal cord. Hemorrhage from rupture of the vessels, or of the structure of the brain, even where paralysis has occurred, is by no means so frequent as in the adult. Nevertheless, in children over two years of age, our author has not unfrequently detected hemorrhage within the substance of the brain after attacks of apoplexy, and, in many cases, persistent paralysis.

When the hyperemia, and effusion of serum or blood, is principally confined to the spine, it constitutes what is called *spinal apoplexy;* the phenomena differ from those of the corresponding lesion of the brain.

"When there is simply turgescence of the vessels and texture of the medulla spinalis, the symptoms that have been observed are, occasionally, convulsions, drowsiness bordering on stupor, lividity of the face, drawing down of the corners of the mouth, and the fixation of the arms firmly against the sides. The symptoms from serous effusion vary somewhat in different cases; thus, in one instance, we may have severe pain in the back, paralysis of the inferior extremities. In another, opisthotonos, difficult deglutition, and coma. In another, violent convulsions, coma, and distortion of the eyes. In another, convulsions, followed by coma, with a permanent clenching of the hands. When blood is effused in the spinal canal, the symptoms are, pain in the back, and general convulsions, or trismus, and convulsions, either tonic or clonic."

Cerebral hemorrhage often occurs with symptoms altogether different from those of ordinary apoplexy, and is generally mistaken for some other lesion of the brain. It is met with in children from one to two years of age, and is rarely seen after the third year.

"The symptoms in the early stage are rather those of encephalic irritation, than of apoplexy; and in the later stage of protracted cases, they differ but little from those of chronic hydrocephalus. The attack sometimes commences with repeated convulsive paroxysms; at other times, with all the phenomena of cerebral inflammation; and in numerous instances, the disease has unquestionably been mistaken for acute hydrocephalus. The child may be affected with vomiting, though in the majority of cases, this symptom is not observed. There is, very generally, in the commencement, severe febrile excitement, with flushed face, hot skin, and a frequent, full, and hard pulse, increased thirst, and loss of appetite. The pulse at first amounting to 100 or 106 in a minute, and soon increasing to 120 or 140, and in the advanced stages of the disease becoming so rapid as scarcely to be counted. Very early in the attack, the patient is affected with slight convulsive movements, particularly of the eyeballs, which are followed by some degree of strabismus. The bowels are in general regular, and the stools natural in appearance. The child is often seen to carry his hands continually, but apparently unconsciously, to some part of the head. There soon occurs a permanent contraction of the feet and hands, followed by convulsions, either tonic or clonic; during which there is an abolition of sensibility and consciousness, and an increased turgescence and coloration of the face; sometimes the convulsions affect the whole of one side of the body, sometimes the upper limbs of one side only; not unfrequently, both sides are affected, but unequally; the convulsive movements being always greater in one than the other. After continuing for a few moments, the convulsions cease, and the patient remains in a state of drowsiness, which increases with the progress of the disease. The febrile symptoms continue throughout the attack, and augment in intensity as death approaches. The convulsive paroxysms occur first after irregular intervals of some length, but become gradually more frequent, until finally, towards the close of fatal cases, they are almost continual, or rather, the patient is affected with a constant tremor, with momentary convulsive paroxysms, during which, the injection of the face is increased, and the pulse and respiration accelerated. In no instance has paralysis been observed during the acute stage. When death takes place, it is generally at the end of eight or twelve days. The occurrence of thoracic inflammation appears, in many cases, to be the cause of death in the acute stage."

The disease may become chronic and continue from eight to thirty months, and would probably continue longer, if, as in the acute stage, accidental causes did not accelerate death. When protracted, the convulsive paroxysms and the febrile symptoms diminish and finally disappear; the cranium enlarges; there is strabismus; dilated pupils; occasionally loss of reason; and in most cases a greater or less impairment of intellect, the child becoming idiotic.

"The appearances upon dissection, when death takes place in the acute stage, are either a simple effusion of bloody serum, or more frequently a bloody serum, containing small, thin, reddish clots of blood, the whole inclosed in a kind of sac, formed by a soft, thin membrane, attached to the lower surface of the arachnoid. The effusion is invariably found within the arachnoid cavity; in the sub-arachnoid cellular membrane, and in the ventricles there generally exists a small quantity of perfectly limpid, or light citrine colored serum. The effusion most commonly occurs upon the surface of both hemispheres. It is sometimes, however, confined to one, and rarely occurs upon the cerebellum. The clots are usually very thin, from two inches and a quarter to two inches and a half in extent, soft, and of a bright color when recent, but becoming brownish or greenish, and more firm the longer the period that has elapsed since the effusion took place. They may exist on a level with the anterior and middle fossæ of the base of the brain; but more generally occur upon the upper surface of the hemispheres. The clots are invariably covered with a reddish, elastic, soft, delicate membrane, but of some degree of firmness, and about one-tenth of an inch in thickness; it may be detached from the surface of the arachnoid in small shreds. This membrane always becomes more elastic and firm the longer the effusion has existed. Most writers refer its production to a mechanical separation of the fibrinous portion of the effused blood, which, deposited on the under surface of the arachnoid, becames gradually organized; the first vestiges of organization presenting themselves about the fifth day after the effusion has occurred.

"The veins of the surface of the hemispheres are occasionally gorged with blood, and the cortical substance of the brain of a very bright reddish grey; the incised surface of the brain becomes studded with numerous bloody points. The convolutions are not sensibly flattened, nor the brain in a state of hypertrophy: it has been supposed to be in some cases even reduced in size, which is, probably, however, a mistake. Not the slightest trace has been discovered in any case, of grey, semi-transparent granulations on the pia mater throughout its extent, nor the least appearance of tubercle, in any part of the brain.

When death occurs at a later period, the clots are found enclosed in a true organized cyst; this adheres intimately to the lower surface of the arachnoid, by means of a very fine, delicate cellular tissue, that is very easily torn, and permits the cyst to be detached entire. The cyst has no attachment to the surface of the brain, excepting by vessels which pass over it to the latter. The contents of the cyst are coagula of blood and a bloody serum, but more generally the last only, which is then always in considerable quantity. The blood is sometimes in the form of soft, red coagula; at others, the cyst contains a brownish, turbid, red serum, in the midst of which float filaments of a soft, somewhat elastic, greyish-red substance, having a close resemblance to fibrine. The parieties of the cyst are thin and transparent; over their surface pass numerous small branches of vessels to the brain. Its cavity is at first single, but subsequently it becomes, by the approximation of its sides, at different points, divided into a number of small cells; there is generally one triangular cavity of an inch or two in length, extending along the falciform process of the dura mater."

The etiology of this form of cerebral hemorrhage is obscure. Age is one of the chief predisposing causes, it being seldom seen in children under one year, and still more seldom in those beyond three. The hemorrhage arises generally from exhalation, rarely from rupture of blood-vessels. Most of the cases observed by Legendre occurred in winter, whereas those noticed by Dr. Condie occurred in the summer, and at least one-third of them were the consequence of insolation.

It is liable to be confounded with tuberculous meningitis. The age of the patient will serve to distinguish them. The latter generally occurs after the sixth year, or at least after the period to which our author restricts cerebral hemorrhage; it is rarely accompanied by the same intense febrile reaction; convulsions are neither so constant nor so frequent; the carpo-pedal contractions are wanting.

In the chronic form it is not easy to distinguish it from chronic hydrocephalus. The latter, however, is congenital or commences soon after birth; the head often acquires an enormous size. The enlargement from chronic cerebral hemorrhage is never so considerable; the affection is never congenital, but commences ordinarily about the tenth month, or at the time of dentition, and is invariably preceded by encephalic symptoms at the time hemorrhage occurs.

From hypertrophy of the brain it may be known by the morbid symptoms in the latter always succeeding or following the enlargement. Hypertrophy is also one of the predisposing causes of cerebral hemorrhage.

In forming a prognosis, the age must be considered. Hemorrhage from rupture of vessels is promptly fatal, but rarely occurs in very young children. When it is produced by simple exhalation, the case is not necessarily fatal; prompt and judicious treatment may cause absorption of the effused fluid. If, however, the acute stage is violent, and the convulsions frequent, or if there is any intercurrent disease, such as pulmonary inflammation, death is almost inevitable.

The treatment of cerebral apoplexy and hemorrhage will be apparent. The abstraction of blood by leeches to the head, or when the child is old enough, bleeding from the arm, is all important. Next purgatives, in which a mercurial should enter; enemata, where purgatives cannot be administered; warm and stimulating pediluvia, whilst cold is applied to the head; blisters behind the ears, or to the back of the neck; with mild diet, plenty of cool fresh air, and cool drinks. The gums should be examined, and incised if they are found swollen. After the occurrence of hemorrhage, the treatment will be nearly the same. Leeches or cups to the head, where there is still undue febrile reaction; purgatives —a combination of calomel, antimony and nitre—warm pediluvia, cold to the head, blisters, &c. In the chronic form the hygienic management is of great importance. "How far," says Dr. C., "we may be able to promote absorption of the effused fluid by the employment of diuretics combined with calomel; iodine externally and internally, and the judicious use of tonics; we must wait for the results of a more extended period to determine."

Allusion has been made to *tubercular meningitis.* Our author gives this term to the disease commonly described under the name of hydrocephalus or dropsy of the brain. We shall not enter into a detail of the symptoms and treatment, which are well known. On examination, in addition to the appearances indicative of meningeal inflammation, the surface of the membranes:

"Is almost invariably found studded with tubercles, varying in size from that of a pin's head to that of a pea; they are generally hard, semi-transparent, and of a grey or yellowish color. Sometimes they present themselves in patches of an inch or more in extent, but, in general, are scattered irregularly over the membrane; they follow the membrane between the convolutions, and are met with, also, imbedded in the grey matter of the brain, where they are often surrounded by a halo of redness, generally connected with an enlarged vessel, ramifying from the pia mater."

*Chronic hydrocephalus*, according to Dr. C., is not a frequent disease in Philadelphia; he has never witnessed a case, either congenital, or developed subsequent to birth. We have mentioned this here for the purpose mainly of introducing the following paragraph in relation to tapping of the brain as a remedy in this affection.

"But the plan in favor of the feasibility, safety, and success of which, the greatest amount of evidence has been adduced, is that of puncturing the brain, drawing off the effused fluid, and preventing its re-accumulation, by pressure applied around the head. The operation consists in passing a small and delicately constructed trochar, into one of the lateral ventricles, and drawing off as much fluid as the powers of the constitution will admit of. The most eligible spot at which the trochar can be introduced, is in the course of the coronal suture, about midway between the crista galli process of the ethmoid bone, and the anterior fontanelle, so that the danger of wounding the corpus striatum is avoided on the one hand, and the longitudinal sinus on the other. The instrument usually penetrates about two inches, and in most cases, the serum discharged is colorless, but occasionally tinged with blood. Sometimes, on withdrawing the trochar, the water will not flow, until a probe has been passed along the canula, to remove portions of brain which block it up. After taking away all the fluid that can be removed, consistently with safety, the head, which should always be steadily compressed, by an assistant, during the operation, may be strapped with adhesive plaster, so as retain its diminished size, and avert the fearful consequences of suddenly removing long-continued pressure from the brain. In no instance, however, has a clearly marked congenital case been permanently benefited; the cases in which the operation has been most successful, are those in which the effusion has manifestly resulted from an inflammatory condition of the brain, and in which cerebral excitement follows the operation."

We have seen one case where this operation was practiced. Convulsions soon followed and the child died in 36 hours. We may mention here that in the acute form, after the active symptoms have heen subdued, and also in the chronic form, the iodide of potassium has been used successfully.

*Cyanosis—morbus cæruleanus—blue disease*, was long supposed to depend upon a patulous state of the foramen-ovale, and the consequent admixture of venous and arterial blood. In the present state of our knowledge on the subject, it cannot be referred to any constant lesion. Dr. C. gives the following summary of the various malformations that have been accompanied by this disease.

"In 53 dissections of patients affected with cyanosis, the foramen ovale was open in 33; in 22 the aorta rose from both ventricles; in 22 the pulmonary artery was contracted; in 14 the ductus arteriosus was open; in 15 the ventricular septum was imperfect; in 5 the pulmonary artery was obliterated; in 4 there was but a single auricle and ventricle; in 4 there was a transposition of the origin of the aorta and pulmonary artery, with an open state of the foramen ovale, and in a few, of the ductus arteriosus, also; in one the aorta was obliterated. The right cavities of the heart are frequently found dilated, hypertrophied, or both. The hypertrophy is always most considerable in cases in which there exists contraction of pulmonary orifice. The total absence of the ventricular septum has been noticed in some cases; in one case, the heart consisted of a single cavity; in another the pulmonary artery arose from both ventricles, sending off the descending aorta; in another, the right auricle opened into the left ventricle, and a free communication existed between the two ventricles and the two auricles; in others the right ventricle was bifid; the arch of the aorta double; the coronary veins opened into the left ventricle; the inferior or the superior cava into the left auricle; the foramen ovale was closed *in the fœtus;* the valves of the heart adhered along their edges, and were consolidated into one substance, leaving only a small central aperture, or else were perforated by numerous holes, or altogether absent. The instances of a single auricle and single ventricle are very numerous. Considerable contraction of the pulmonary artery, particularly at its origin, is a very common lesion; in a few cases, the pulmonary artery has been found completely impervious; in one case the artery was entirely absent, the bronchial arteries appearing to supply its place; and in another, the aorta terminated near the heart, in a *cul de sac.* In one case there was an entire absence of the right lung, the septum between the ventricles of the heart was imperfect, the foramen ovale and ductus arteriosis were both open: the aorta communicated with both ventricles; the pulmonary artery was imperforate at the base of the heart; the lung being supplied with blood by the arterial duct; the child lived six weeks."

Our author then proceeds:

"Sufficient evidence is thus afforded that abnormal communications between the cavities of the heart, particularly the auricles, by the non-closure of the foramen ovale; and various malformations of that organ, and of the main arterial and venous trunks, occur, in perhaps the majority of cases of cyanosis. By far the most frequent, however, is the non-closure of the foramen ovale; how far this is capable of producing the disease under consideration, is a question, however, that is yet by no means satisfactorily answered. The mixture of the arterial and venous blood, which is supposed to result from the free communication between the two auricles, is evidently inadequate to explain the blue color of the skin, inasmuch as no such discoloration takes place in the fœtus; while a communication exists in the majority of infants for many days after birth, without a single symptom indicative of cyanosis, being present. The fœtal openings in the heart, and the communication between the aorta and pulmonary artery, by means of the ductus arteriosus, are not usually obliterated before the eighth day subsequent to birth, and have even been found open as late as the third week, in infants who have died of diseases totally unconnected with any disturbance of the respiratory apparatus, in the slightest degree resembling that which occurs in cyanosis. It has been shown, in numerous instances, that the foramen ovale has remained open for a long period, without the occurrence of cyanosis; and it has been denied that any admixture of venous and arterial blood can take place, even with a free opening between the two auricles, from non-closure of the foramen, so long as the communicating cavities are of equal strength, or provided all the orifices are free. Cyanosis has occurred in numerous instances in which no abnormal condition of the heart, or main arterial trunks, existed; and it has been absent in cases in which extensive malformation of the heart was present. It is very improbable indeed that, so long as the lungs continue to perform fully their office, and a sufficient supply of oxygenated blood is regularly distributed to the different parts of the body—even although this should be mixed with a portion of venous blood, the phenomena of cyanosis can occur: these depend, evidently, upon a disturbance of the function of the lungs, by which the due arterialization of any portion of the blood is prevented; or upon such a malformation of the heart, or abnormal distribution of the main arterial trunks, that venous, instead of arterial blood, is distributed to the organs. It may, therefore, exist without any abnormal condition of the heart, if from any cause, the blood in passing through the lungs, is prevented from undergoing the change which it is the office of these organs to effect in it; while even with extensive malformation in the circulating organ, it will not occur, so long as the lungs perform their functions freely. Cyanosis is common in many of the pulmonary affections occurring during infancy; it is also invariably present in that condition of the lungs, resulting from imperfect respiration, denominated *atelectasis.* It has been found associated with enlargement of the heart, contraction of the pulmonary arteries, and extensive 'tuberculous hardening' of the lungs; with contraction of the diameter of the glottis and trachea; with extensive disease of the brain or spinal marrow, without any abnormal changes in the heart, lungs, or great vessels, and with severe abdominal inflammations. It is also asserted that many cases of cyanosis, owe their origin, in the adult female, to some disturbance of the menstrual function, especially to sudden suppression of the discharge."

There are some other chapters which we intended to notice, but the length to which our quotations have extended, warns us to stop. The extracts we have made will however be sufficient for our purpose. In copying, we have omitted the numerous citations of authorities with which the author has interlarded and disfigured his text, and which apparently belong to all nations, kindreds, and tongues. Chiefly, though, we believe, does he affect the German. Many of his formulæ (which we have omitted—we never could recollect one in our life, and, for that reason perhaps, have no great opinion of the man who can) are derived from German authors, and of course muriate of ammonia and hyosciamus mingle in them largely and frequently. It is probable the former is too little employed by us as an internal remedy. The latter is certainly quite a favorite with our author. He prescribes it on almost all occasions; in truth his preference for it is decidedly *hobby-horsical*, as Mr. Shandy would phrase it.

We have said nothing yet of the first part of the volume, in which the author has brought together many things of importance touching the *hygienic management of children*, *the peculiarities of organization and function in infancy and childhood*, *the pathology of infancy*, and *the semeiology of the diseases of infancy and childhood.* This is by no means the least interesting or the least valuable part of the treatise. In fact it very properly and necessarily precedes the discussion of diseases and their remedies. Here again there is a copious citation of authorities—the German being duly favored of course. We must not be understood as finding fault with our author's preference for the medical literature of that nation. He has as much right to draw from its copious stores in this department, as had Campbell, Coleridge, Carlyle, Scott, Shelley, Bulwer, and many others, to take from other departments, and he has had the honesty, which the first two had not, to acknowledge his indebtedness. Nor must we be understood as objecting to his candor in telling us on what grounds he has poached, or in what region he has levied black-mail. It was no doubt wise in him to do so; for without such precaution he might have found it as dangerous to draw from some of these sources as poor Maginn thought it would be 'to steal from Sam Rogers.'

We commend this portion of the volume especially to the perusal of the young practitioner. In all probability the diseases of childhood will be the very first with which he has to deal. It does seem a little strange, to be sure, that what would puzzle and does puzzle the oldest and wisest heads in the profession, is expected to be fully understood by the youngest and the least wise. Yet so far as our observation has extended, certainly so far as our personal experience goes, it is to the young practitioner that the care of the *babies* falls, whilst that of the precious parent devolves upon the grey-beards of the profession. Perhaps it is because in this, as in other things, less value is placed on the common and familiar (in the West, it is not the soil only which is productive)—perhaps it is owing to the general prevalence of the sentiment uttered by Napoleon on occasion of the accouchement of his royal consort—perhaps it is on the principle of *lucus a non lucendo*—perhaps it is ordained for wise purposes and provides for the absence of the *prudential restraint* so strenuously urged by certain philanthropists—we do not know; but the fact is unquestionable; let the young physician prepare himself. C.

